# CSIOVDB: a microarray gene expression database of epithelial ovarian cancer subtype

**DOI:** 10.18632/oncotarget.5983

**Published:** 2015-11-07

**Authors:** Tuan Zea Tan, He Yang, Jieru Ye, Jeffrey Low, Mahesh Choolani, David Shao Peng Tan, Jean-Paul Thiery, Ruby Yun-Ju Huang

**Affiliations:** ^1^ Cancer Science Institute of Singapore, National University of Singapore, Center for Translational Medicine, Singapore 117599; ^2^ Department of Obstetrics and Gynecology, National University Health System, Singapore 119228; ^3^ Department of Haematology-Oncology, National University Hospital, Singapore 119074; ^4^ Institute of Molecular and Cell Biology, A*STAR, Proteos, Singapore 138673; ^5^ Department of Biochemistry, Yong Loo Lin School of Medicine, National University of Singapore, Singapore 117596; ^6^ Department of Anatomy, Yong Loo Lin School of Medicine, National University of Singapore, Singapore 117596

**Keywords:** ovarian cancer, microarray gene expression, molecular subtype, database

## Abstract

Databases pertaining to various diseases provide valuable resources on particular genes of interest but lack the molecular subtype and epithelial-mesenchymal transition status. CSIOVDB is a transcriptomic microarray database of 3,431 human ovarian cancers, including carcinoma of the ovary, fallopian tube, and peritoneum, and metastasis to the ovary. The database also comprises stroma and ovarian surface epithelium from normal ovary tissue, as well as over 400 early-stage ovarian cancers. This unique database presents the molecular subtype and epithelial-mesenchymal transition status for each ovarian cancer sample, with major ovarian cancer histologies (clear cell, endometrioid, mucinous, low-grade serous, serous) represented. Clinico-pathological parameters available include tumor grade, surgical debulking status, clinical response and age. The database has 1,868 and 1,516 samples with information pertaining to overall and disease-free survival rates, respectively. The database also provides integration with the copy number, DNA methylation and mutation data from TCGA. CSIOVDB seeks to provide a resource for biomarker and therapeutic target exploration for ovarian cancer research.

## INTRODUCTION

Every year, it is estimated that 238,700 women will develop ovarian cancer worldwide. Epithelial ovarian cancer (EOC) is the fifth-most common cause of female cancer death, with an estimated 151,900 deaths [1]. Even though the 5-year survival rates for localized, regional, and distant ovarian cancer are 91%, 72% and 27%, respectively, 61% of cases are presented at a stage when the disease is already widely metastatic [2], explaining the high mortality rate for this disease [3]. The majority of EOC patients respond well to first line platinum-based chemotherapy but about two-thirds of patients will eventually relapse with disease regardless of the initial clinical response [3]. Patients with recurrent EOC may initially respond to further chemotherapeutic agents but eventually develop chemoresistant disease and succumb to their illness.

At least 5 different histological subtypes of EOC exist and may reflect the clinical heterogeneity of this disease [4] in terms of chemotherapeutic response and outcome. Recently, it is becoming increasingly clear from the molecular analysis of EOC that this is also a molecularly heterogeneous disease [5–7]. While the relative clinical significance of these individual EOC molecular subtypes, as defined by high-throughput transcriptomics, remains unclear, recent data suggest that the gene expression profiles of EOC may have predictive value in determining patient benefit from targeted therapeutic agents such as bevacizumab (Avastin^®^) in frontline therapy [8]. Specifically, the mesenchymal/C1 [5–7] and Stem-A/Proliferative/C5 [5–7] subtypes were demonstrated to respond better to bevacizumab-containing regimen in ICON7 trials, with improvements in progression-free survival of 8.1 and 10.1 months, respectively [9]. Likewise, our group has also previously described that the Stem-A molecular subtype of EOC is sensitive to microtubule-targeted compounds such as vincristine and vinorelbine. On the other hand, the epithelial-mesenchymal transition (EMT) spectrum based on gene expression has also been described in EOC, where differential responses for epithelial-like and mesenchymal-like ovarian cancers have been reported; for example, mesenchymal ovarian cancer is reported to be more sensitive to cisplatin and benefits from a paclitaxel-containing treatment regimen [10, 11]. These studies suggest the clinical relevance of ovarian cancer molecular subtyping, and the potential to identify targeted therapies utilizing the molecular subtyping or EMT status.

Databases such as cBioportal [12, 13] and KMplotter [14] offer valuable resources to investigate genes of interest in various diseases, including ovarian cancer. In addition, OvMark [15], a database dedicated to investigate mRNA and miRNA expression in ovarian cancer provided tremendous insight into progression of the disease. However, none provide an indication of molecular subtype or EMT status. In this work, we built a transcriptomics database of human ovarian cancer, referred to as CSIOVDB (Ovarian cancer database of Cancer Science Institute Singapore; http://csibio.nus.edu.sg/CSIOVDB/CSIOVDB.html), which is furnished with molecular subtype and EMT information. Through this database, we seek to provide a complementary resource for gene expression profiling in ovarian cancer, particularly, the differential expression of molecular subtypes and its correlation with the EMT status. By delineating these transcriptomic subtypes and EMT status of EOC, it is envisaged that our database will facilitate further strategies to explore and guide targeted therapeutic approaches in this challenging disease.

## RESULTS

### Profiles of CSIOVDB

**Figure 1:**
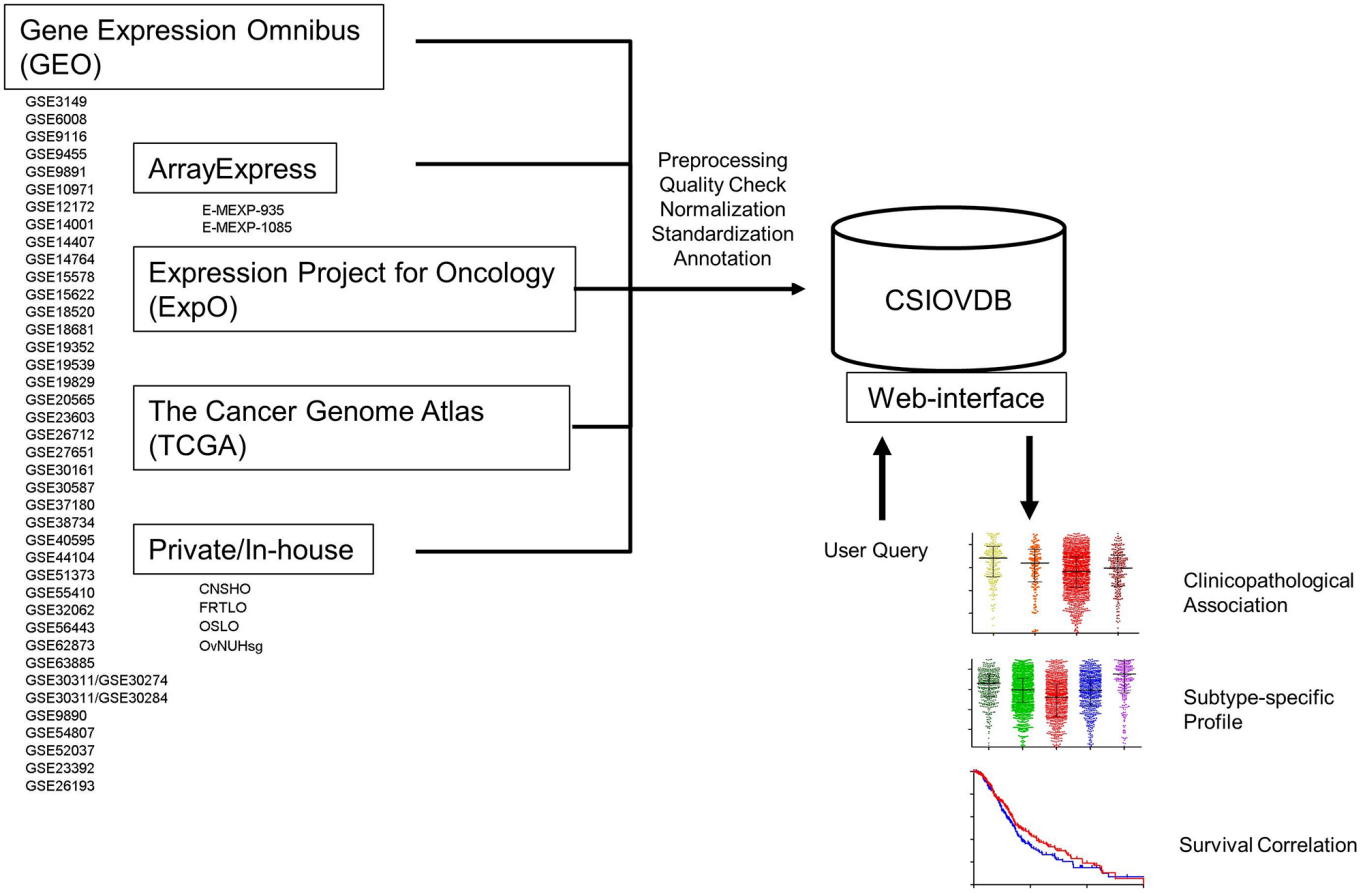
Structure of CSIOVDB CSIOVDB housed data of human ovarian carcinoma from GEO, ArrayExpress, TCGA, ExpO, and private/in-house. The data from different cohorts were compiled, and subjected to quality check, RMA normalization, and standardization to remove batch effect. Clinical annotation was extracted from the data repository or original publication. User can query a gene of interest to CSIOVDB using a browser. Output of the query includes clinical association of the gene expression, gene expression profiles in different histologies, molecular subtypes, as well as survival correlation. Molecular subtype-specific correlation is also provided.

**Figure 2:**
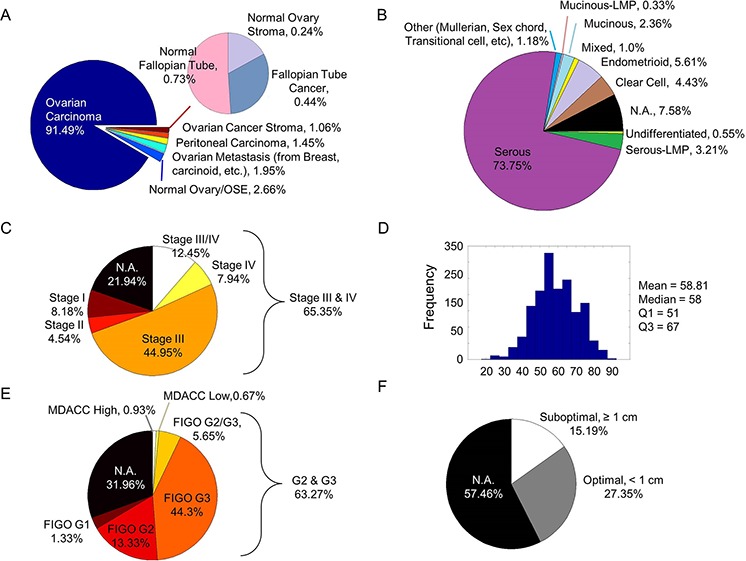
Clinico-pathological profiles of CSIOVDB Pie charts or histogram showing distribution of various parameters in CSIOVDB: **A.** disease state, **B.** WHO histology, **C.** FIGO staging, **D.** age, **E.** FIGO grading or MDACC two-tier grading, and **F.** surgery debulking status. Abbreviation: N.A., not available.

CSIOVDB comprises 3,431 microarray samples from 48 cohorts of private, in-house and public human ovarian cancer datasets (Materials and Methods; Figure 1; Suppl. Figure 1; Suppl. Table 1). The database contains 3,261 unique samples of mainly primary and metastatic ovarian cancers (91.49%), as well as fallopian tube carcinoma (0.44%), peritoneal carcinoma (1.45%), metastasis to the ovary from elsewhere (1.95%), and ovarian cancer stroma (1.065). Non-cancerous samples constitute 3.63% of CSIOVDB and include normal ovarian surface epithelium (2.66%), normal ovary stroma (0.24%), and normal fallopian tube (0.73%; Figure 2A). Epithelial ovarian cancer is the main component of CSIOVDB; non-epithelial ovarian cancer, such as ovarian germ cell tumors, sex-cord stromal, and sarcoma (Figure 2B) comprise less than 1% of the database. Note, however, that CSIOVDB does not mirror the actual frequency of ovarian cancer, where non-epithelial ovarian cancer accounts for 10% of all ovarian cancers [16]. In terms of morphology, high-grade serous ovarian cancer is the most prevalent (73.75%) in CSIOVDB; this closely follows the 70% prevalence of high-grade serous ovarian cancer [4]. Ovarian cancers of other histologies, however, are slightly under-represented in CSIOVDB (CSIOVDB% vs reported%): mucinous (2.36% vs 3%), endometrioid (5.61% vs 10%), clear cell (4.43% vs 10%), and serous with low malignant potential (3.21% vs 5%) [4]. Late- (III & IV), and early-stage (I & II) tumor samples represent 65.35% and 12.72%, respectively, of the database (Figure 2C). The median age of the CSIOVDB samples is 58 years (Figure 2D). No menopausal information is available. Ovarian cancer grading is assessed either by FIGO (64.4%) or by the University of Texas M. D. Anderson Cancer Center [17] system (1.6%). High-grade ovarian cancers form the majority of CSIOVDB (63.27%; Figure 2E). Optimal (27.35%) and suboptimal (15.19%) surgical debulking status is also noted (Figure 2F), as this status is associated with ovarian cancer survival [18]. Surprisingly, whereas the surgical debulking status is associated with survival, this parameter does not contribute significantly to the molecular differences in ovarian cancer (Suppl. Figure 6D). Overall and disease-free survival data are available for 1,868 and 1,516 samples, respectively, with a median overall survival of 31.67 months and median disease-free survival of 17.09 months (Suppl. Table 2). Finally, molecular subtyping and EMT scores are provided in CSIOVDB. The database comprises 11.75% of ovarian cancer with an Epi-A subtype, 29.04% with Epi-B, 29.01% with Mes, 19.2% with Stem-A and 8.23% with Stem-B ovarian cancer; this spread of tumors mirrors the distribution of previous analyses [5] (Suppl. Figure 6A). Thus, overall, CSIOVDB represents a large and diverse collection of ovarian cancer that could be useful for assessing a gene of interest.

### Features of CSIOVDB

**Figure 3:**
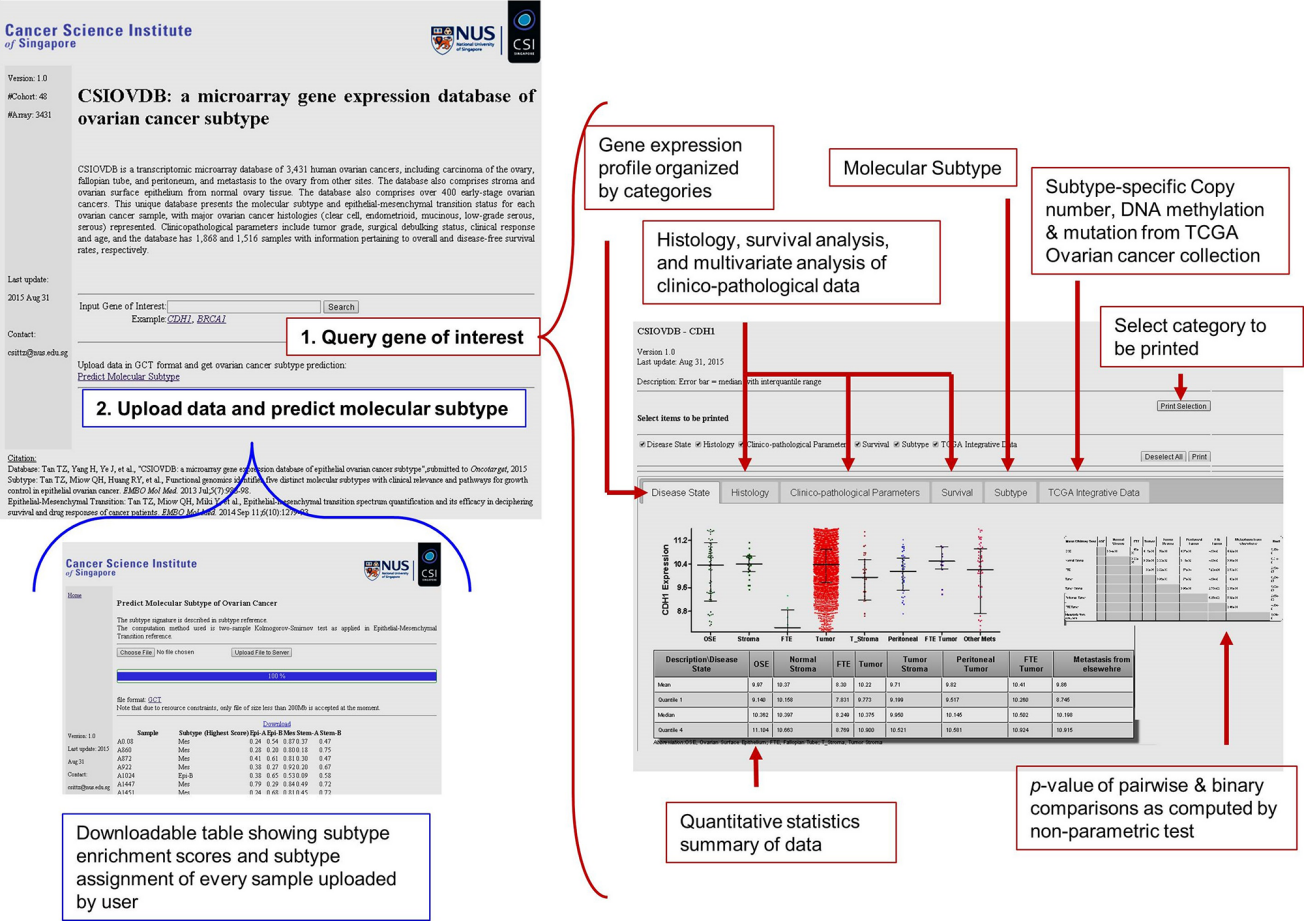
Screenshot of CSIOVDB Snapshot of CSIOVDB showing functions available on CSIOVDB. Firstly, query a gene of interest; and secondly, predict ovarian cancer subtype. Red or blue boxes indicate the features available on the main page: queried gene page and the page of predicting ovarian cancer subtype.

A screenshot of CSIOVDB is given in Figure 3 summarizing the features available. At the main page, there are two functions available: first, users can choose to query a gene of interest to CSIOVDB. At the result page of gene queried, the expression profiles of gene of interest are organized into different categories: disease state, histology, clinico-pathological parameters, and molecular subtype. In addition, molecular subtype-specific copy number, mutation, and DNA methylation profile from TCGA of the queried gene are provided in a separate tab. Quantitative statistics such as mean, median, upper and lower quantiles are available. Pairwise and binary significance assessments were performed using Mann-Whitney, Spearman correlation coefficient, or log-rank test. Multivariate Cox regression of queried gene and clinico-pathological parameters was also performed. User can select a subset of categories to be printed. Second, users can upload dataset for computation of ovarian molecular subtype. The computation method is based on two-sample Kolmogorov-Smirnov test and a subtype signature as described in previous work [5]. Computation of EMT score can be requested through email or from [10].

### Querying a gene of interest

**Figure 4:**
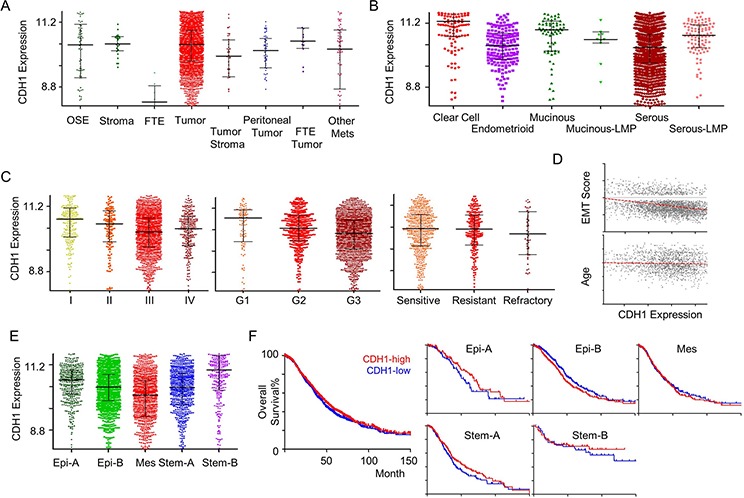
*CDH1* gene expression in ovarian cancer An example of CSIOVDB outputs for a gene queried. Gene expression profiles of *CDH1* in ovarian cancer disease state **A.** histology **B.** FIGO stage **C.** Left panel FIGO grade C. Middle panel. clinical response C. Right panel epithelial-mesenchymal transition (EMT) score **D.** Upper panel age D. Lower panel molecular subtype **E.** and overall survival **F.** Left panel overall survival within subtype F. Right panel. Median expression was used to define CDH1-high and CDH1-low groups. Linear regression line fit is shown in red in (D). Abbreviation: OSE, ovarian surface epithelium; FTE, fallopian tube epithelium; Mets, metastasis; LMP, low-malignant potential; Epi, epithelial; Mes, mesenchymal; Stem, stem-like. Error bar is median ± quantile.

CSIOVDB seeks to provide users with the expression profiles of certain genes of interest relevant to ovarian cancer; in particular, the molecular subtype distribution and subtype-specific outcomes in terms of overall survival and disease-free (progression- and recurrence-free) survival. Figure 4 and Table 1 show a subset of outputs available from CSIOVDB when the gene *CDH1* is queried (http://csibio.nus.edu.sg/CSIOVDB/pages/CSIOVDB_CDH1.html). *CDH1* is an epithelial marker that codes for E-cadherin. The loss of E-cadherin expression has been linked to cancer progression and metastasis [19] and shown to display a differential expression profile in ovarian cancer (Figure 4). Interestingly, there is no significant difference in *CDH1* expression between ovarian cancer and normal ovarian surface epithelium (*p* = 0.415; Figure 4A), and this adheres to the previous findings that ovarian surface epithelium has both mesenchymal and epithelial features [20]. Neither peritoneal (*p* = 0.0177) nor fallopian tube carcinoma (*p* = 0.459) shows differential expression of *CDH1* with ovarian carcinoma. As expected, ovarian cancer stroma, which is more mesenchymal-like, has a significantly lower *CDH1* level than its carcinoma counterpart (*p* = 0.004). From a histological perspective, high-grade serous and endometrioid ovarian cancers have the lowest *CDH1* expression (Figure 4B). The less-aggressive serous carcinoma with low malignant potential has significantly higher *CDH1* expression compared with high-grade serous ovarian cancer (*p* = 4.12E-08). Also not surprisingly, the more metastatic and aggressive late-stage (*p* = 1.44E-12) and high-grade (*p* = 4.42E-05) ovarian cancer have significantly lower *CDH1* expression (Figure 4C); however, there is no difference in *CDH1* expression for clinical response (Figure 4C). Since *CDH1* is an epithelial marker, it displays a negative correlation with EMT score (*Rho* = −0.32; Figure 4D). No correlation was observed between *CDH1* expression and age (*Rho* = −0.067; Figure 4D). Importantly, *CDH1* was found to be lowest in the Mes subtype (*p* = 7.15E-38; Figure 4E), which is enriched with metastatic ovarian cancers [5] and supports the conjecture that a loss of *CDH1* promotes metastasis [19]. Despite evidence for the loss of *CDH1* in tumors with a Mes subtype, as well as the association between a Mes subtype and metastasis, our database shows no correlation for *CDH1* with overall survival for any of the subtypes (Figure 4E, 4F). This is likely due to the fact that *CDH1* is expressed in Stem-A, another poor survival subtype (Figure 4E, Suppl. Figure 6B).

**Table 1:** Multivariate Cox regression analysis of ovarian cancer overall survival

Prognostic Factor	Cox Coefficient	*p*-value
Stage (I, II vs III, IV)	1.40	1.0E-5
Grade (G1 vs G2, G3)	0.65	0.0931
Surgical Debulking (optimal vs suboptimal)	0.2	0.0373
Histology (Non-serous vs serous)	1.29	0.0105
Age (< 55 vs ≥ 55)	0.16	0.0777
CDH1	−0.05	0.5966

This example of the data obtained as an output from CSIOVDB also demonstrates the range of descriptive statistics provided (mean, median, quantiles and statistical significance evaluations). Furthermore, a multivariate Cox regression analysis of *CDH1* gene expression levels and known ovarian cancer prognostic factors, such as stage, grade, surgical debulking status, histology and age, is provided (Table 1). Importantly, CSIOVDB provided not only gene expression profiles of molecular subtypes, but also subtype-specific survival outcomes.

## DISCUSSION

CSIOVDB is a transcriptomics database of human ovarian cancer comprising 3,431 microarray data from 48 cohorts. Each sample is coupled with histology, molecular subtype and EMT information to enable the user to explore and investigate genes of interest. To the best of our knowledge, this is the first database that integrates molecular subtype and EMT with ovarian cancer. This unique feature of CSIOVDB allows interrogation of subtype-specific expression as well as survival profiles. It is our hope that the database will provide a complementary resource to existing general [12–14] or databases specifically dedicated for ovarian cancer [15] for the investigation of clinical associations for genes of interest and, more importantly, for the localization and assessment of potential biomarkers or therapeutic targets for ovarian cancer. The previously determined preferential responses of mesenchymal-like ovarian cancer to paclitaxel [10], and platinum [11], the chemosensitivity to vincristine and vinorelbine in Stem-A ovarian cancer [5], and the chemosensitivity to bevacizumab in mesenchymal and Stem-A (proliferative) ovarian cancer [9] all suggest the feasibility of targeted therapeutics for ovarian cancer and, by extension, the utility of CSIOVDB.

However, it is important to note that ovarian cancer is an extremely heterogeneous disease [21]—95% of ovarian cancers are clonally heterogeneous and many have four or more subclones [22]. Not surprisingly, therefore, most ovarian cancer exhibit properties of multiple subtypes [23]. There are as many as 82% of the TCGA and 42% of the Mayo ovarian cancer cohorts displayed properties of at least two subtypes [24]. Thus, ovarian cancer treatment regimens may require a multi-agent approach, targeting the different subclones that exhibit diverse subtypes; it is plausible that targeting one subclone will only allow another to take over [25]. Adding to this complexity, studies have shown that the molecular subtype of a tumor may change post-chemotherapy [26], prompting the need for a continuous subtype re-assessment during the course of chemotherapy. In CSIOVDB, samples are assigned to the strongest phenotypic subtype (as a subtype gene expression signature [5]). Given that each subtype is represented by a sufficiently large number of samples, the influence of sample heterogeneity on subtype gene expression may be mitigated.

Aside from heterogeneity within a sample, a further caveat of using CSIOVDB is that the database is built based on public data from different repositories contributed by various authors and laboratories. While we have made every endeavor to limit the effect of a center-related batch effect, factors [27] such as reagents, protocols, procedures, elapsed time from sample collection, therapy regimen, and many others, could not be accounted for because of a lack of data. Thus, the data obtained through CSIOVDB should be viewed as a preliminary analysis, and users are urged to exercise due diligence in validating their findings.

On a side note, to ensure the relevance of CSIOVDB, we plan to annually update CSIOVDB to include new publicly available data as well as update of clinical and annotation data. In addition, we intend to replicate the process of building CSIOVDB to other cancers and allow users to query a gene of interest in multiple cancers and molecular subtypes.

## MATERIALS AND METHODS

### Eligibility criteria

As our purpose was to compile a database of broader generalizations and larger sample size, we adopted less stringent eligibility criteria [28]. Three private, one in-house and 44 publicly available microarray ovarian cancer datasets from Gene Omnibus (GEO; http://www.ncbi.nlm.nih.gov/gds), ArrayExpress (http://www.ebi.ac.uk/arrayexpress/), Expression Project for Oncology (ExpO; http://www.intgen.org/), and The Cancel Genome Atlas (TCGA; http://cancergenome.nih.gov/) were downloaded by Jan 2015 (Figure 1; Suppl. Table 1). Only datasets obtained using Affymetrix Microarrays HG-U133A (16.38%), HG-U133A2 (2.88%), HG-HT-U133A (17.25%), HG-U133-Plus2 (49.2%), and human gene 1.0 ST (14.29%) were used. These datasets are inclusive of primary and metastatic ovarian cancers, fallopian tube carcinoma, peritoneal carcinoma, ovarian cancer stroma, and normal ovarian surface epithelium, fallopian tube, and stroma tissues (Figure 2). No limit was imposed on the race, pre-treatment history or medical condition, stage, grade, or histology of the disease.

### National University Hospital cohort

Frozen archival EOC samples from Department of Obstetrics & Gynecology, National University Hospital of Singapore were collected from 2006 to 2014. Frozen tumor samples were kept frozen at all times prior to evaluation. Each frozen tumor sample was pounded to a fine powder in liquid nitrogen using a pre-chilled mortar and pestle, and the powdered sample collected into a pre-chilled microfuge tube. Samples were homogenized in Trizol (Life Technologies, Carlsbad, CA) using a sterile 1-ml syringe and a 21-G hypodermic needle (BD Precision, Oxford, AL). After homogenization, RNA was purified using a Qiagen miRNeasy kit, as per manufacturer's protocol (Hilden, Germany). RNA sample quality was determined by Eukaryote Total RNA Nano Series II, 2100 Bioanalyzer (Agilent Technologies, Santa Clara, CA). RNA samples with a RIN value above 6.5 were used for the Affymetrix GeneChip^®^ Human Gene 1.0 ST Array (Affymetrix, Inc., Santa Clara, CA). Data has been deposited in GEO with the accession GSE69207.

### Clinico-pathological parameters

All clinical and pathological information were extracted either from ArrayExpress and GEO, or from publications associated with the data. Samples without sufficient information are flagged as ‘Not Available’. In some categories, abstraction or simplification was performed to have a sizeable group; for example, the histopathology of ovarian carcinoma, as classified according to the World Health Organization (WHO; http://www.who.int), is restricted to (high-grade) serous, borderline/low-malignant potential (LMP) serous, mucinous, endometrioid, and clear cell. Other less-prevalent ovarian cancers, such as Brenner, Signet ring cell, sex cord-gonadal stromal, and mixed Mullerian tumors, and ovarian cancers with mixed histologies are grouped as ‘other’ and ‘mixed’, respectively; no distinction is made for the International Federation of Gynecology and Obstetrics (FIGO; http://www.figo.org) group within each stage (e.g. stages IA, IB, IC are grouped as stage I). Grading of ovarian cancer was conducted using FIGO or the two-tier grading system proposed by the University of Texas M. D. Anderson Cancer Center [17]; these two grading systems usually show good correlation [17]. The optimal surgical debulking status is defined as having residual tumor of less than 1 cm. Clinical response is defined by either response evaluation criteria in solid tumors (RECIST) version 1.0 and above, or the serum level of CA-125; these two tests also show comparable results [29]. Pathological response is also available in some of the ovarian cancer samples, where pathological complete response is defined as no residual carcinoma or no residual invasive tumor. For simplicity, we categorized clinical response into ‘sensitive’ (RECIST complete response; pathological complete responder), ‘resistant’ (RECIST partial response, stable disease; pathological non-complete responder), and ‘refractory’ (RECIST progressive disease; pathological non-responder). Overall survival was computed by the difference between the date of last follow-up (or date of death) and the date of diagnosis, regardless of the cause. Disease-free survival encompasses progression-, local and distant recurrence-free survival.

### Preprocessing of affymetrix expression data

A total of 3,431 arrays corresponding to 3,261 unique patients were collected. Prior to normalization, quality control was performed on the Affymetrix chips using R version 3.1.2 (2014–10-31) and Bioconductor packages (affy version 1.42.3, affyQCReport version 1.42) for 3′IVT arrays (HG-U133 series), or Affymetrix Power Tools version 1.15.2 for human exon array (human gene 1.0 ST). Details of R session information can be found in the Supplementary Information. Quality metrics and the following criteria were analyzed: average perfect-match (PM) intensity, background, scale factor, GAPDH 3′:5′ ratio, β-actin 3′:5′ ratio, area under the curve (AUC) of positive versus negative controls, relative log expression median, relative log expression inter-quantile range (Suppl. Figure 2). All chips passed at least one of the criteria, and hence, none of the samples was discarded.

For the post-quality check, the data from the 48 cohorts was combined and normalized using frozen robust multichip average (fRMA) [30] version 1.16. Annotation details for each platform, as required by fRMA, are given in the R session information in the Supplementary Information. Three datasets—U133A-U133P2-Gene1.0ST, U133P2-Gene1.0ST, and Gene1.0ST—were compiled to obtain probes/genes unique to each platform (Suppl. Figure 3). Probe matching was performed based on Affymetrix HG-U133-Plus2 to human gene 1.0 ST good- and perfect-matched probe-sets (the product sheet is available at http://www.affymetrix.com/support/technical/byproduct.affx?product=hugene-1_0-st-v1). In the case where multiple-to-one or one-to-multiple probes matched, only those probe-sets with highest intensities were kept. Annotation of the probes is based on the Affymetrix annotation version na34. The combined and normalized data were subsequently standardized using ComBat [31] to remove any batch effect by the cohorts and the centers. The influence of batch effect was assessed for clinico-pathological parameters and potential confounding factors: cohort, processing batch, centers and platform, pre- and post-ComBat standardization (Suppl. Figure 4A). A Kruskal-Wallis test of the first 45 principal components (>90% variance) and the parameters/factors indicates that the batch effect due to cohort, processing batch, center and platform were minimized post-standardization without removing differences due to clinico-pathological parameters (Suppl. Figure 4A; Suppl. Info). Inter-sample correlations and subtype concordance analyses of JPKO (GSE30311) samples available on both Affymetrix U133-Plus2 and human gene 1.0 ST provide additional support that the batch effect was not overwhelming in CSIOVDB (Suppl. Figures 4B & 4C; Suppl. Info).

### Ovarian cancer molecular subtype and epithelial-mesenchymal transition (EMT) status

Gene expression values of ovarian cancer molecular subtype signatures [5] were extracted from the standardized dataset and subjected to clustering using Bioconductor ConcensusClusterPlus version 1.18. Parameters chosen were hierarchical clustering with agglomerative average linkage, with Euclidean distance and a sub-sampling ratio of 0.8 for 1000 iterations. The condition *K*_max_ was set to 5 to assign each sample to one of the ovarian cancer molecular subtypes: Epithelial-A (Epi-A), Epi-B, Mesenchymal (Mes), Stem-like (Stem)-A, or Stem-B (Suppl. Info). EMT scores for each sample were computed using an ovarian cancer-specific EMT signature and two-sample Kolmogorov-Smirnov test, as described previously [10].

### TCGA ovarian cancer data integration

Level-3 and level-2 data of ovarian cancer SNP array, Illumina human 27K DNA methylation array and exome-sequencing were respectively downloaded from TCGA data portal (https://tcga-data.nci.nih.gov/tcga/) on August 31, 2015. Samples were matched by TCGA patient ID and samples with molecular subtype available (computed in this study) were used for analyses.

### Multivariate cox regression analysis

Multivariate survival analyses of known ovarian cancer prognostic factors (stage, grade, age, histology and surgical debulking status) and gene expression were computed using Cox regression on 987 (overall survival) or 778 (disease-free survival) ovarian cancer samples with all clinico-pathological data available. In the multivariate Cox regression analysis, the factors and gene expression parameters are converted to binary states: stage is categorized as early (I, II) or late (III, IV); grade is categorized as low (G1) or high (G2, G3); age is categorized as young (< 55) or old (≥ 55); histology is categorized as non-serous or serous; and gene expression is categorized as low (< median expression) or high (≥ median expression).

### Statistical analysis

Statistical analyses were conducted using Matlab ^®^ R2012a version 7.14.0.739, and statistics toolbox version 8.0 (MathWorks; Natick, MA). Statistical significance of differential expression was evaluated using either Kruskal-Wallis or Mann-Whitney *U*-test. A Spearman correlation coefficient test was applied to assess significance of correlation. Kaplan-Meier analyses were conducted using GraphPad Prism ^®^ version 5.04 (GraphPad Software; La Jolla, CA). Statistical significance of the Kaplan-Meier analysis was calculated by log-rank test.

## SUPPLEMENTARY DATA, MATERIALS, METHODS, FIGURES AND TABLES




