# Supplementary material for: CSIOVDB: a microarray gene expression database of epithelial ovarian cancer subtype
Source: Oncotarget. 2015 Nov 7;6(41):43843–52. doi: 10.18632/oncotarget.5983 (PMC4791271; doi:10.18632/oncotarget.5983)
Supplement: Supplementary file 1 [file oncotarget-06-43843-s001.pdf]

# SUPPLEMENTARY DATA

## MATERIALS AND METHODS

### Assessment of batch effect

To assess the influence of batch effect on the various clinicopathological parameters and potential confounding factors, such as fRMA processing batch, cohort, center and platform, principal component analysis was performed pre- and post-ComBat standardization (Suppl. Figure 4A). The first to 45$^{th}$ principal components, corresponding to more than 93.6% variance, were used for assessment. A Kruskal-Wallis test was conducted for each discrete parameter/factor (fRMA processing batch, cohort, stage, grade, surgical debulking status, disease state, histology, clinical response, center, and platform), whereas a Spearman Correlation Coefficient test was performed for continuous parameters (age and array scandate). The procedures were repeated for U133A-U133P2-Gene1.0ST, U133P2-Gene1.0ST, and Gene1.0ST. From Suppl. Figure 4A, the potential batch effect due to confounding factors (fRMA processing batch, cohort, center, platform) and array scan date were effectively reduced post-ComBat standardization while still retaining the variance due to clinicopathological parameters. Of note, the surgical debulking status does not contribute significantly to the variance of ovarian cancer, implying that the underlying variance between tumors in CSIOVDB was mainly due to biological difference and less affected by the skill of surgeon.

To verify that the CSIOVDB is minimally affected by batch effects, we performed inter-sample correlations for a Japanese cohort, JPKO (GSE30311), which has the same samples hybridized on Affymetrix U133-Plus2 and human gene 1.0 ST (Suppl. Figure 4B). Correlations were significantly higher in the same-sample than in the inter-sample Spearman Correlation *Rho* ($p$ = 1.93E-6), indicating that the batch effects due to microarray platform, scan date, and cohort were not overwhelming. A correlation of JPKO samples with random non-JPKO samples were also conducted to ensure the high same-sample correlation observed was not due to random chance (Suppl. Figure 4B, lower panel). As expected, the correlation of JPKO with random non-JPKO samples was significantly lower ($p$ = 1.51E-6) than that of same-sample correlation.

Upon confirming the good correlation for gene expression profiles, we tested the subtype calling for the JPKO samples that were hybridized on both Affymetrix U133-Plus2 and human gene 1.0 ST arrays (Suppl. Figure 4C). We found that the subtype calls were largely concordant (97.14%) between the two platforms, aside from two Epi-A samples that were classified as Stem-B. Of note, Epi-A and Stem-B subtypes are similar and more related as compared with the other subtypes [1]. There are eight bridging samples between different hybridization runs, as well as the two platforms, and we observed 100% concordance for these eight bridging samples for subtype assignment. This suggests that the influence of batch effect from scan date and platform were not overpowering the underlying biological difference in CSIOVDB. Taken together, these analyses provide evidence for the utility of CSIOVDB in analyses of biological differences among ovarian cancer samples.

### Molecular subtype of ovarian cancer

The molecular subtypes of ovarian cancer were previously proposed [1]: Epithelial (Epi)-A, Epi-B, Mesenchymal (Mes), Stem-like (Stem)-A, and Stem-B. Applying the same procedure and subtype signature as before [1], CSIOVDB ovarian cancer samples were clustered into five subgroups in a manner highly concordant (>75%) with previous analyses [1] (Suppl. Figure 5A). The CSIOVDB heatmap of subtype signature is given in Suppl. Figure 5B. Concordance was also observed for highly expressed subtype markers SOX11, HMGA2 in Stem-A; HLAs, immunoglobulins in Epi-B; collagens, and other mesenchymal markers in Mes [1]. The clinical features of the subtypes also concurred with previous findings [1] (Suppl. Figure 6). The frequency distribution of the subtypes is also similar to that found before [1], with Epi-B and Mes subtypes forming the majority in the cohort (Suppl. Figure 6A). In accordance with previous findings [1], Mes and Stem-A subtypes have the worse prognosis (Suppl. Figure 6B); Stem-B is enriched with non-high grade serous ovarian cancer (Suppl. Figure 6C); Epi-A is enriched in low-grade and early-stage ovarian cancer (Suppl. Figure 6D); and Stem-A is enriched in older ovarian cancer patients (Suppl. Figure 6D). Of note, echoing the finding in the principal component analysis in Suppl. Figure 4A, we found no subtype distribution difference associated with the surgical debulking status (Suppl. Figure 6D). Overall, the subtypes that have been previously reported were consistent and reproduced in CSIOVDB.

### R session information

R version 3.1.2 (2014–10-31)
Platform: x86_64-w64-mingw32/x64 (64-bit)
locale:
[1] LC_COLLATE=English_Singapore.1252 LC_CTYPE=English_Singapore.1252

LC_MONETARY=English_Singapore.1252 LC_NUMERIC=C

LC_TIME=English_Singapore.1252

attached base packages:

[1] parallel stats graphics grDevices utils datasets methods base

other attached packages:

[1] oligo_1.28.3 Biostrings_2.32.1 XVector_0.4.0 IRanges_1.22.10 oligoClasses_1.26.0

[5] hugene.1.0.st.v1frmavecs_1.0.0 hgu133plus2frmavecs_1.3.0 frma_1.16.0

[8] affyQCReport_1.42.0 lattice_0.20–29 affy_1.42.3 Biobase_2.24.0

[12] BiocGenerics_0.10.0 ConsensusClusterPlus_1.18.0

loaded via a namespace (and not attached):

[1] affxparser_1.36.0 affyio_1.32.0 affyPLM_1.40.1 annotate_1.42.1

[5] AnnotationDbi_1.26.1 BiocInstaller_1.14.3 bit_1.1–12 cluster_1.15.3

[9] codetools_0.2–9 DBI_0.3.1 ff_2.2–13 foreach_1.4.2 gcrma_2.36.0

[14] genefilter_1.46.1 GenomeInfoDb_1.0.2 GenomicRanges_1.16.4 grid_3.1.2

[18] iterators_1.0.7 MASS_7.3–35 preprocessCore_1.26.1 RColorBrewer_1.1–2

[22] RSQLite_1.0.0 simpleaffy_2.40.0 splines_3.1.2 stats4_3.1.2 survival_2.37–7

[27] XML_3.98–1.1 xtable_1.7–4 zlibbioc_1.10.0

## REFERENCES

1. Tan TZ, Miow QH, Huang RY, Wong MK, Ye J, Lau JA, Wu MC, Bin Abdul Hadi LH, Soong R, Choolani M, Davidson B, Nesland JM, Wang LZ, Matsumura N, Mandai M, Konishi I, et al. Functional genomics identifies five distinct molecular subtypes with clinical relevance and pathways for growth control in epithelial ovarian cancer. EMBO molecular medicine. 2013; 5:983–998.

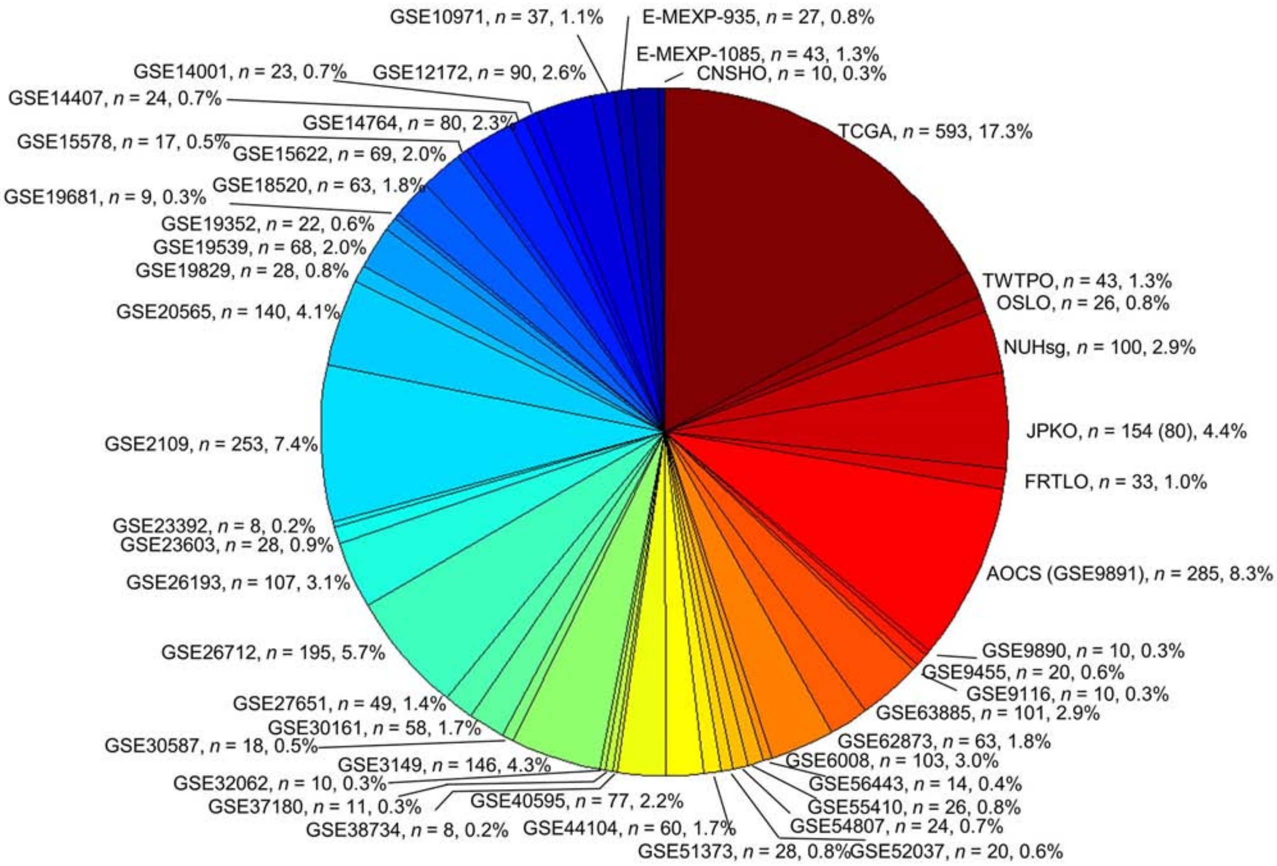


**Supplementary Figure S1: Cohorts in CSIOVDB.** Pie chart of 48 human ovarian carcinoma cohorts used in building CSIOVDB. The database features 3,431 arrays corresponding to 3,261 unique patients. The original ArrayExpress or GEO IDs, the number of samples, and the relative percentage of each cohort in CSIOVDB are given.

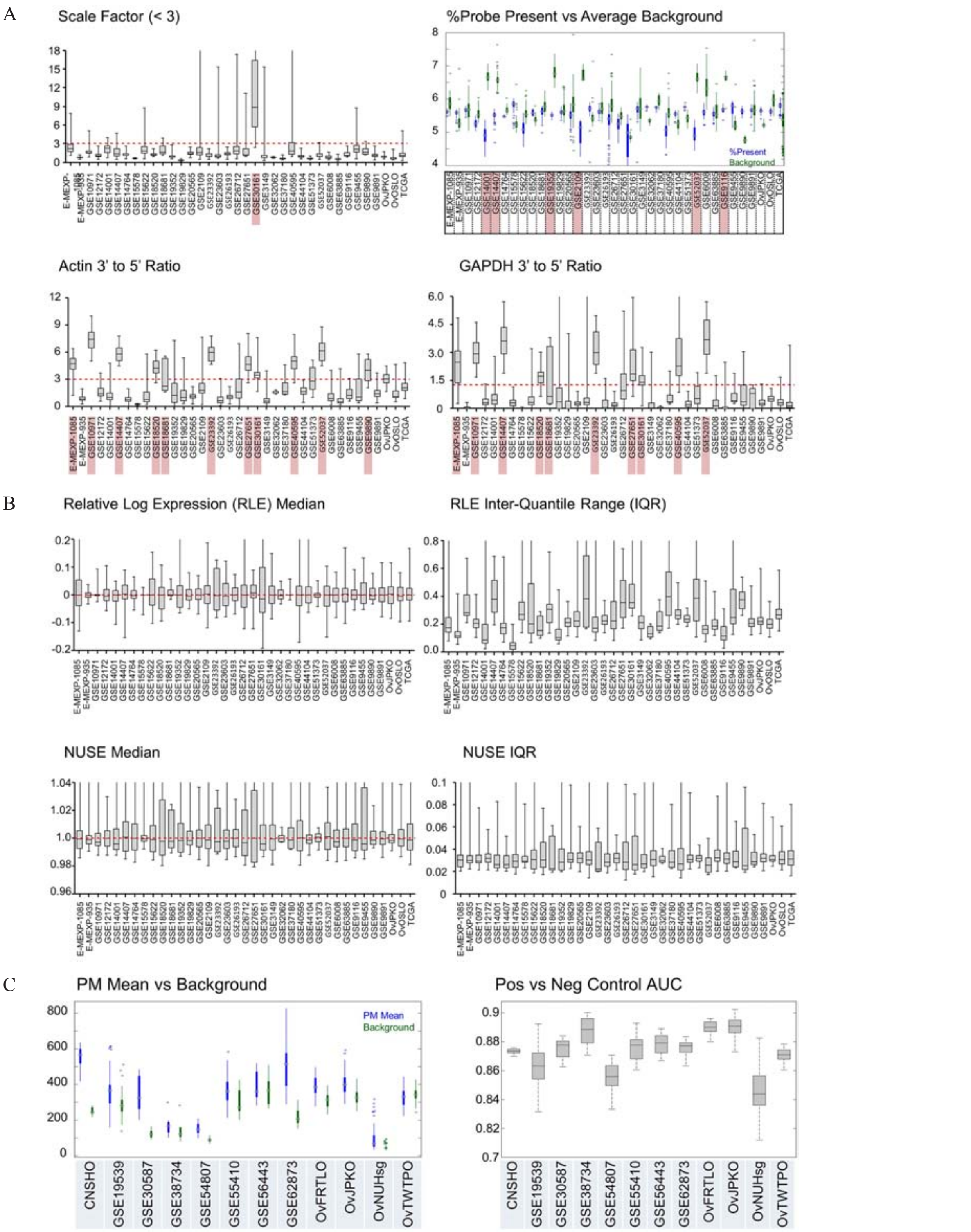


**Supplementary Figure S2: Array quality check result.** Quality metrics boxplots of microarrays on Affymetrix U133A, A2 and Plus2 **A. & B.** and human gene 1.0 ST **C.** used in CSIOVDB. The top and bottom whiskers indicate the maximum and minimum values, respectively, whereas the box indicates the upper quantile, median and lower quantile. *Abbreviation*: PM, perfect match; Pos, positive; Neg, negative; AUC, area under the curve. Cohorts with samples not meeting recommended criteria are highlighted in red.

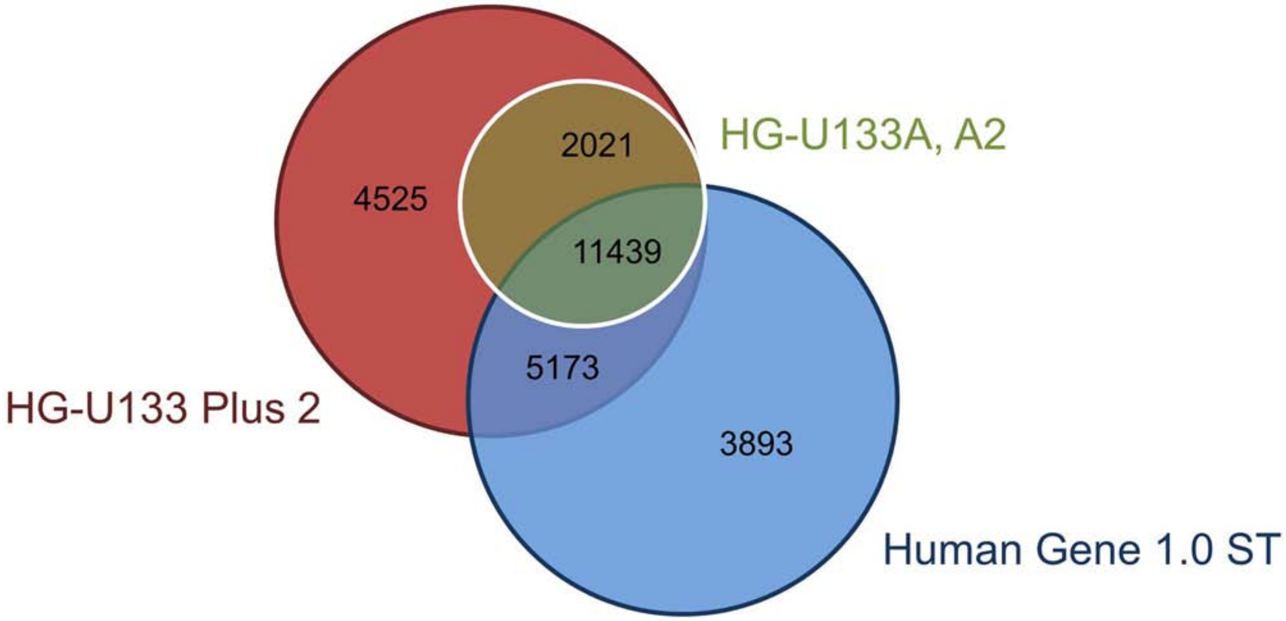


**Supplementary Figure S3: Overlap of probe-sets between platforms.** Venn diagram showing the overlap of probe-sets between Affymetrix U133A, A2, Plus2 and human gene 1.0 ST.

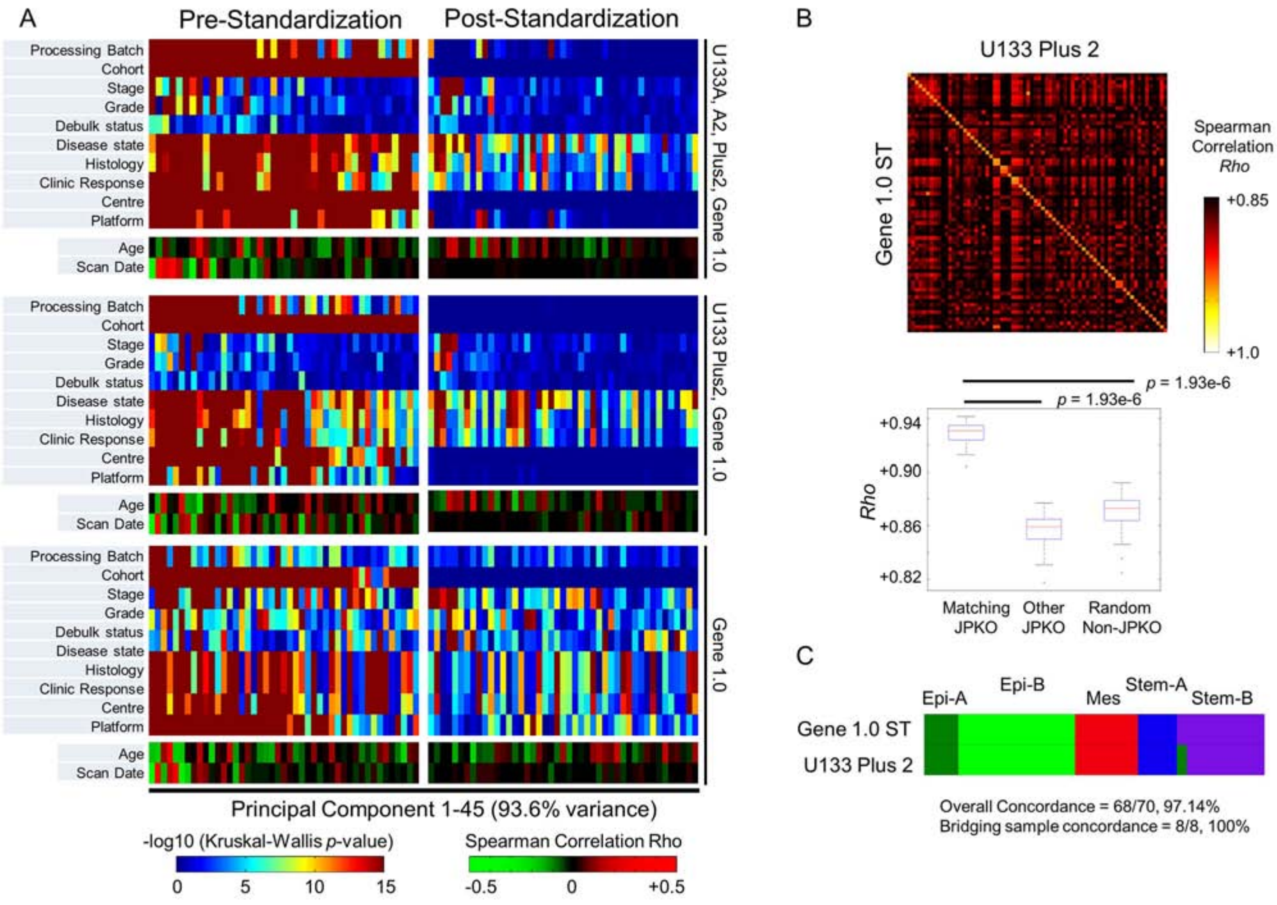


**Supplementary Figure S4: Removing batch effect due to cohorts and centers.** **A.** Pre- (left panel) and post-standardization (right panel) heatmap of –log10 Kruskal-Wallis test *p*-values (maroon = more significant, blue = less significant) assessing variance due to fRMA processing batch, cohort, stage, grade, surgery debulking status, disease state, histology, clinical response, center, and platform, as well as heatmap of –log10 Spearman correlation coefficient test *Rho* (red/green = correlated, black = not correlated) assessing age and array scan date using the first 45 principal components (corresponding to > 93% of data variance). **B.** Upper panel. Heatmap of inter-sample Spearman correlation *Rho* (white = more correlated, black = less correlated) using JPKO cohort on Affymetrix U133-Plus2 and on human gene 1.0 ST. Lower panel. Boxplot of Spearman correlation *Rho* between a sample on Affymetrix U133-Plus2 and its matching sample on human gene 1.0 ST, non-matching samples from JPKO cohort, and random samples from non-JPKO cohort (averaged over 100 permutations). **C.** Colormap of predicted ovarian molecular subtype of matching samples from Affymetrix U133-Plus2 and human gene 1.0 ST. Color code: Epithelial (Epi)-A, dark green; Epi-B, light green; Mesenchymal (Mes), red; Stem-like (Stem)-A, blue; Stem-B, purple.

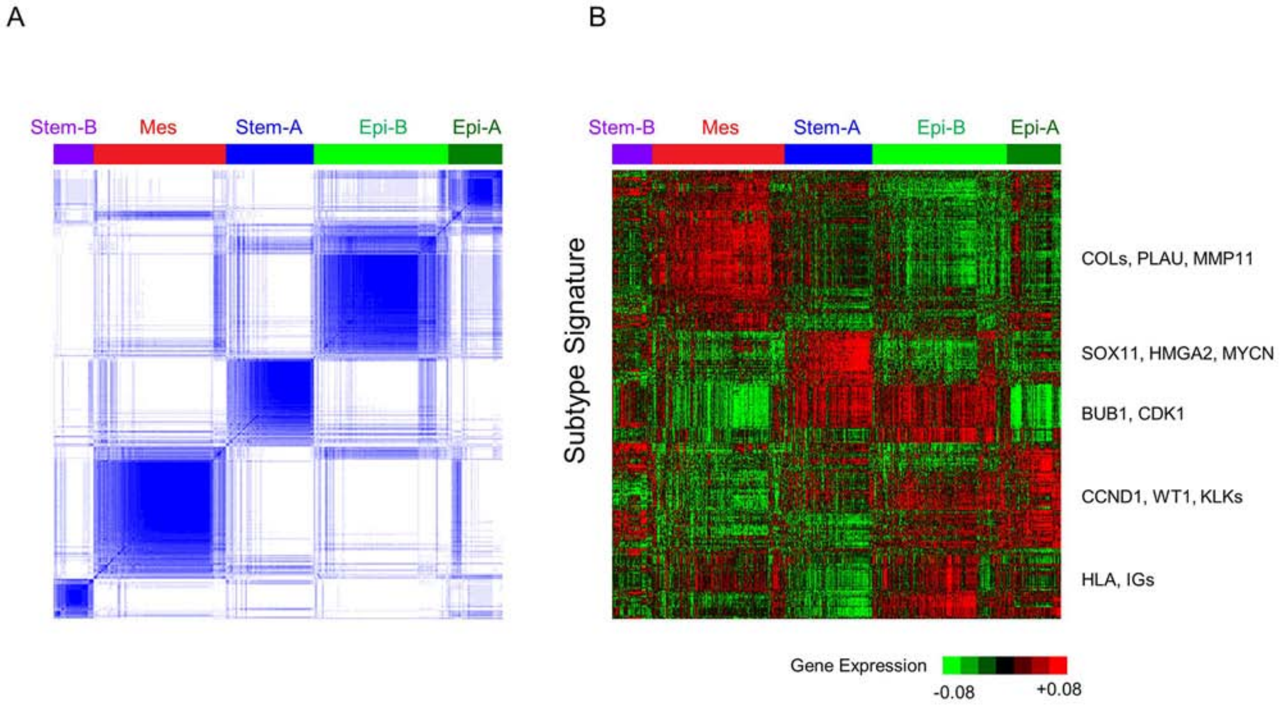


**Supplementary Figure S5: Consensus clustering of CSIOVDB samples.** Results of consensus hierarchical clustering on 3,431 arrays on CSIOVDB. The samples were aligned based on the clusters identified by hierarchical clustering. The similarity matrix of the samples is given in **A.** and the heatmap of ovarian cancer subtype signatures [1] (red = high expression, green = low expression) is given in **B.**

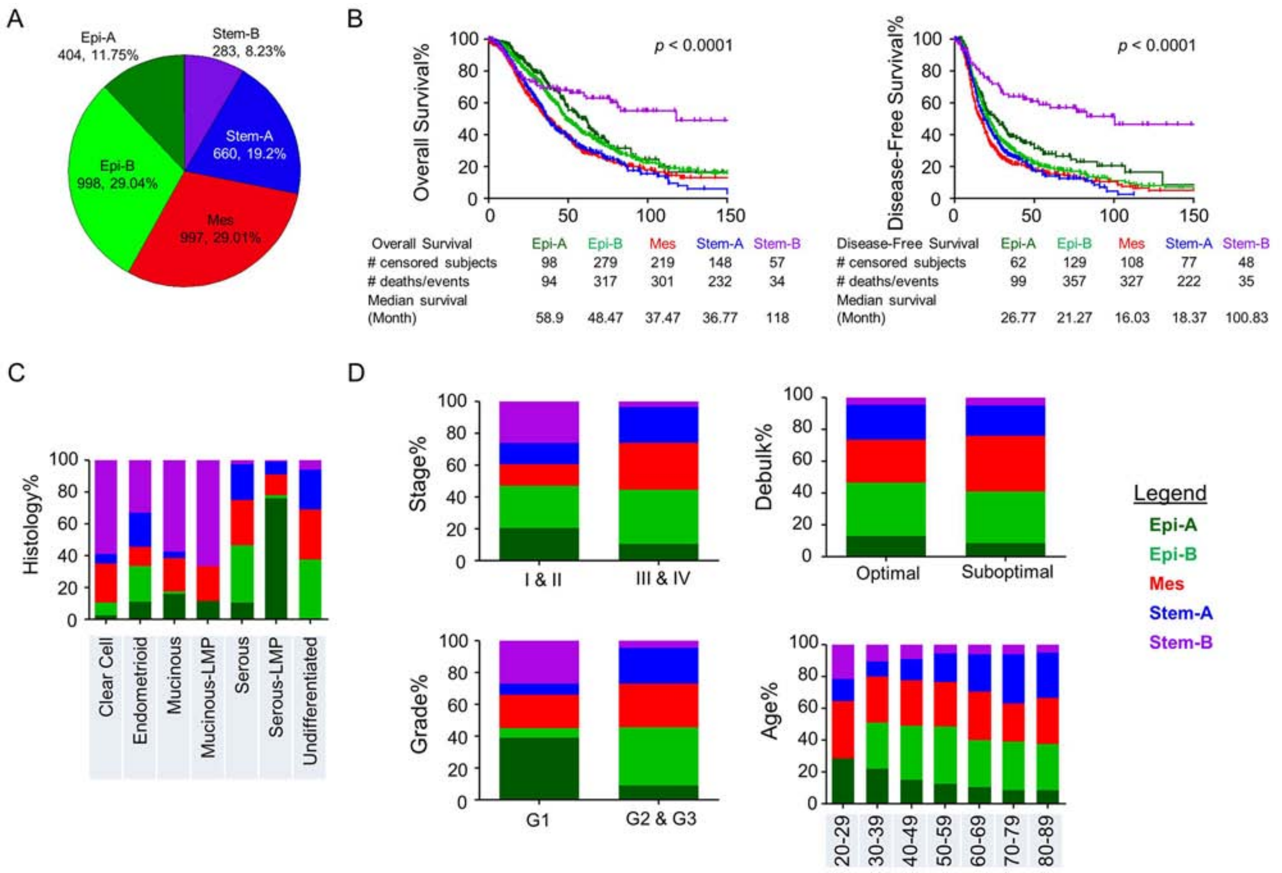


**Supplementary Figure S6: Clinico-pathological characteristics of ovarian cancer subtypes. A.** Pie charts of ovarian cancer subtypes. **B.** Left panel. Overall survival curves of the subtypes ($n = 1,868$). Right panel. Disease-free survival (DFS) curves of the subtypes ($n = 1,516$). DFS includes progression- and recurrence-free survival. Median survival in months for each subtype is given in the table below the curve. The *p*-value is computed by log-rank test. Bar plots show the distribution of subtypes in histology **C.** stage **D.** upper left panel grade D; lower left panel surgery debulking status D; upper right panel and age D; lower right panel. Color code: Epithelial (Epi)-A, dark green; Epi-B, light green; Mesenchymal (Mes), red; Stem-like (Stem)-A, blue; Stem-B, purple.

**Supplementary Table S1: List of datasets used for ovarian cancer database compilation.**

**Supplementary Table S2: Summary of clinicopathological parameters for CSIOVDB**

| Category | Description | #sample |
|---|---|---|
| Total | — | 3431 |
| Normal-Ovary/OSE | Disease state | 90 |
| Normal-Stroma | Disease state | 8 |
| Normal-Fallopian Tube | Disease state | 24 |
| Tumor | Disease state | 3095 |
| Tumor-Stroma | Disease state | 36 |
| Peritonium | Disease state | 49 |
| FallopianTube | Disease state | 15 |
| Other | Disease state | 66 |
| Overall Survival data | Survival outcome | 1868 |
| Overall Survival.Month | Survival outcome | 31.67 |
| Overall Survival.Event | Survival outcome | 995 |
| Disease-Free Survival data | Survival outcome | 1516 |
| Disease-Free Survival.Month | Survival outcome | 17.09 |
| Disease-Free Survival.Event | Survival outcome | 1087 |
| Stage I | Staging | 281 |
| Stage II | Staging | 156 |
| Stage III | Staging | 1545 |
| Stage IV | Staging | 273 |
| Stage III/IV | Staging | 428 |
| Stage Unknown | Staging | 754 |
| Grade 1 | Grading | 109 |
| Grade 2 | Grading | 458 |
| Grade 3 | Grading | 1522 |
| Grade 2/3 | Grading | 194 |
| Grade MDACC Low | Grading | 23 |
| Grade MDACC High | Grading | 32 |
| Grade Unknown | Grading | 1098 |
| Optimal | Debulking Status | 940 |
| Suboptimal | Debulking Status | 522 |
| Debulk Status Unknown | Debulking Status | 1975 |
| Histology Unknown | Histology | 250 |
| Clear Cell | Histology | 146 |
| Endometrioid | Histology | 185 |
| Mixed | Histology | 33 |

(*Continued*)

| Category | Description | #sample |
|---|---|---|
| Mucinous | Histology | 78 |
| Mucinous-Low Malignant Potential | Histology | 11 |
| Other | Histology | 39 |
| Serous | Histology | 2433 |
| Serous-Low Malignant Potential | Histology | 106 |
| Undifferentiated | Histology | 18 |
| Clinic Response Unknown | Clinical Response | 2528 |
| Complete Response/Responder (CR) | Clinical Response | 531 |
| Non-Responder (NR) | Clinical Response | 52 |
| Progressive Disease (PD) | Clinical Response | 49 |
| Partial Response (PR) | Clinical Response | 109 |
| Refractory | Clinical Response | 8 |
| Resistant | Clinical Response | 50 |
| Stable Disease (SD) | Clinical Response | 39 |
| Sensitive | Clinical Response | 71 |
| Age Unknown | Age | 1728 |
| Median Age | Age | 58 |
| U133A | Platform | 563 |
| U133A2 | Platform | 99 |
| HTU133A | Platform | 593 |
| U133P2 | Platform | 1691 |
| Gene st1.0 | Platform | 491 |
