# Supplementary material for: CSIOVDB: a microarray gene expression database of epithelial ovarian cancer subtype
Source: Oncotarget. 2015 Nov 7;6(41):43843–52. doi: 10.18632/oncotarget.5983 (PMC4791271; doi:10.18632/oncotarget.5983)
Supplement: Supplementary file 2 [file oncotarget-06-43843-s002.pdf]

Supplementary Table 1: List of datasets used for ovarian cancer database compilation

SN	ArrayExpress/ GEO ID	Project Title	Number of Tumor Samples + Normal	Platform	Overall Survival	Disease-Free Survival	Stage	Tumor Site	Age	Histology	LMP	Grade	Micro-dissection	Pubmed ID	Reference
1	GSE3149	Duke Cohort	146	U133A	Yes	No	Yes	No	Yes	Serous	No	Yes	No	16273092	Bild AH, Yao G, Chang JT, Wang Q et al. 2006 Nature **439** 353-7
2	---	CNSHO Cohort	10	Gene st1.0	No	No	Yes	No	No	Serous/Endo/Clear/Muci	No	No	No	---	---
3	E-MEXP-935	Transcription profiling of human ovarian cancer tumor samples to investigate heterogeneity	27	U133P2	No	No	Yes	No	No	Serous/Endo/Clear/Muci	No	No	No	17367315	Jochumsen KM, Tan Q, Hølund B, Kruse TA, Mouritsen L, Thompson RG...
4	E-MEXP-1085	Transcription profiling of human ovarian tumors from the MALOVA (MALignant OVArian) cancer study	43	U133P2	No	No	No	No	No	Serous	No	No	No	19823056	Jochumsen KM, Tan Q, Høgdall EV, Høgdall C et al. 2009 Int J Gynecol Cancer. **19** 1205-13
5	GSE2109	Expression Project for Oncology (expO)	253	U133P2	No	No	Yes	Yes	Yes	Serous/Endo/Clear/Muci	Yes	Yes	No	---	---
6	GSE6008	Human ovarian tumors and normal ovaries	99+4	U133A	No	No	Yes	No	No	Serous/Endo/Clear/Muci	No	Yes	No	16452189	Hendrix ND, Wu R, Kuick R, Schwartz DR et al. 2006 Cancer Res **66** 1354-62.
7	GSE9116	Differential gene expression in primary ovarian tumors from depressed and non-depressed patients	10	U133A	No	No	No	No	No	Serous	No	No	No	18550328	Lutgendorf SK, DeGeest K, Sung CY, Arevalo JM et al. Depression, social support, and beta-adrenergic transcription control in human ovarian cancer. 2009 Brain Behav Immun **23**(2):176-83.
8	GSE9455	Pre-treatment expression data from patients recruited to the paclitaxel arm of the CTCR-OV01 study	20	U133A2	No	No	No	No	No	NA	No	No	No	18068629	Ahmed AA, Mills AD, Ibrahim AE, Temple J et al. The extracellular matrix protein TGFBI induces microtubule stabilization and sensitizes ovarian cancers to paclitaxel. 2007 Cancer Cell **12**(6):514-27
9	GSE9891	Expression profile of 285 ovarian tumor samples	285	U133P2	Yes	Yes	Yes	Yes	Yes	Serous/Endo	Yes	Yes	No	18698038	Tothill RW, Tinker AV, George J, Brown R et al. 2008 Clin Cancer Res **14** 5198-208.
10	GSE10971	Gene expression data from non-malignant fallopian tube epithelium and high grade serous carcinoma.	13	U133P2	No	No	No	Yes	Yes	Serous	No	No	Yes	18593983	Tone AA, Begley H, Sharma M, Murphy J et al. 2008 Clin Cancer Res **14** 4067-78.
11	GSE12172	Common activation of RAS_MAPK pathway in serous LMP tumors	90	U133P2	No	No	Yes	Yes	No	Serous	Yes	Yes	No	19010816	Anglesio MS, Arnold JM, George J, Tinker AV et al. 2008 Mol Cancer Res **6** 1678-90.
12	GSE14001	PAX2: A Potential Biomarker for Low Malignant Potential Ovarian Tumors and Low-Grade Serous Ovarian Carcinomas	20	U133P2	No	No	No	No	No	Serous	No	Yes	No	19525924	Tung CS, Mok SC, Tsang YT, Zu Z et al. 2009 Mod Pathol **22** 1243-50.
13	GSE14407	Ovarian Cancer gene expression profiling identifies the surface of the ovary as a stem cell niche	12	U133P2	No	No	Yes	Yes	Yes	Serous	No	Yes	Yes	20040092	Bowen NJ, Walker LD, Matyunina LV, Logani S et al. 2009 BMC Med Genomics **2** 71.
14	GSE14764	A Prognostic Gene Expression Index in Ovarian Cancer	80	U133A	Yes	Yes	Yes	No	No	Serous/Endo	No	Yes	No	19294737	Denkert C, Budczies J, Darb-Esfahani S, Gyorffy B et al. 2009 J Pathol **218** 273-80.
15	GSE15578	Expression Data from Ovarian Surface Kinome	4	U133P2	No	No	No	No	No	Serous	No	No	No	19956396	Pejovic T, Pande NT, Mori M, Mhawech-Fauceglia P, et al. 2009 Transl Oncol **2** 341-9
16	GSE15622	Expression data from the CTCR-OV01 study	37	U133A2	Yes	Yes	Yes	No	No	Serous	No	Yes	No	18068629	Ahmed AA, Mills AD, Ibrahim AE, Temple J, Blenkiron C, Vias M, Massie CE, Iyer NG, McGeoch A, Crawford R, Nicke B, Downward J, Swanton C, Bell SD, Earl HM, Laskey RA, Caldas C, Brenton JD. The extracellular matrix protein TGFBI induces microtubule stabilization and sensitizes ovarian cancers to paclitaxel. 2007 Cancer Cell **12**(6):514-27.
17	GSE18520	Whole-genome oligonucleotide expression analysis of papillary serous ovarian adenocarcinomas	53	U133P2	Yes	No	Yes	No	No	Serous	No	Yes	Yes	19962670	Mok SC, Bonome T, Vathipadiekal V, Bell A et al. 2009 Cancer Cell **16** 521-32.
18	GSE18681	Gene expression profile of ascites cell samples from patients with advanced ovarian cancer	9	U133P2	No	No	No	No	No	NA	No	No	No	22065722	Kulbe H, Chakravarty P, Leinster DA, Charles KA, Kwong J, Thompson RG, Coward JI, Schioppa T, Robinson SC, Gallagher WM, Galletta L; Australian Ovarian Cancer Study Group, Salako MA, Smyth JF, Hagemann T, Brennan DJ, Bowtell DD, Balkwill FR. A dynamic inflammatory cytokine network in the human ovarian cancer microenvironment. 2012 Cancer Res **72**(1):66-75.
19	GSE19352	Activation of phosphatidylcholine-cycle enzymes in human epithelial ovarian cancer cells	20	U133P2	No	No	No	No	Yes	Serous/Other	No	Yes	No	20179205	Iorio E, Ricci A, Bagnoli M, Pisanu ME et al. 2010 Cancer Res **70** 2126-35.
20	GSE19539	Identification of Novel Oncogene Loci in Ovarian Cancer through Integrated Copy Number and Expression Analysis	68	Gene st1.0	No	No	Yes	No	Yes	Serous/Endo/Clear/Muci	No	Yes	No	20386695	Ramakrishna M, Williams LH, Boyle SE, Bearfoot JL, Sridhar A, Speed TP, Gorringe KL, Campbell IG. Identification of candidate growth promoting genes in ovarian cancer through integrated copy number and expression analysis. 2010 PLoS One **5**(4):e9983.
21	GSE19829	A gene expression profile of BRCAness that is associated with outcome in ovarian cancer	28	U133P2	Yes	Yes	Yes	No	Yes	Serous	No	Yes	No	20547991	Konstantinopoulos PA, Spentzos D, Karlan BY, Taniguchi T et al. 2010 J Clin Oncol. **28** 3555-61.
22	GSE20565	Primary and secondary ovarian tumors	140	U133P2	No	No	Yes	No	No	Serous/Endo/Clear/Muci	No	Yes	No	20492709	Meyniel JP, Cottu PH, Decraene C, Stern MH et al. 2010 BMC Cancer **10** 222.
23	GSE23603	Gene expression in ovarian cancer	28	U133A	Yes	No	Yes	No	No	Serous	No	Yes	No	24849418	Marchion DC, Cottrill HM, Xiong Y, Chen N, Bicaku E, Fulp WJ, Bansal N, Chon HS, Stickles XB, Kamath SG, Hakam A, Li L, Su D, Moreno C, Judson PL, Berchuck A, Wenham RM, Apte SM, Gonzalez-Bosquet J, Bloom GC, Eschrich SA, Sebti S, Chen DT, Lancaster JM. BAD phosphorylation determines ovarian cancer chemosensitivity and patient survival. 2011 Clin Cancer Res. **17**(19):6356-66.
24	GSE26712	A Gene Signature Predicting for Survival in Suboptimally Debulked Patients with Ovarian Cancer	185 + 10	U133A	Yes	No	Yes	No	No	Serous	No	Yes	No	18593951	Bonome T, Levine DA, Shih J, Randonovich M et al. 2008 Cancer Res. 2008 **68** 5478-86
25	GSE27651	The Anterior Gradient Homolog 3 (AGR3) Gene Is Associated with Differentiation and Survival in Ovarian Cancer	43	U133P2	No	No	No	No	No	Serous	Yes	Yes	Yes	21451362	King ER, Tung CS, Tsang YT, Zu Z et al. 2011 Am J Surg Pathol. **35** 904-12
26	GSE30161	Genomic Multivariate Predictors of Response to Adjuvant Chemotherapy in Ovarian Carcinoma: Predicting Platinum Resistance	58	U133P2	Yes	Yes	Yes	No	Yes	Serous/Endo/Clear/Muci	No	Yes	No	22348014	Ferriss JS, Kim Y, Duska L, Birrer M, Levine DA, Moskaluk C, Theodorescu D, Lee JK. Multi-gene expression predictors of single drug responses to adjuvant chemotherapy in ovarian carcinoma: predicting platinum resistance. 2012 PLoS One. **7**(2):e30550
27	GSE30587	Adaptation During Ovarian Cancer Metastasis Reveals a Predictive Gene Signature	18	Gene st1.0	No	No	No	No	No	Serous	No	No	No	24732363	Brodsky AS, Fischer A, Miller DH, Vang S, MacLaughlan S, Wu HT, Yu J, Steinhoff M, Collins C, Smith PJ, Raphael BJ, Brard L. Expression profiling of primary and metastatic ovarian tumors reveals differences indicative of aggressive disease. 2014 PLoS One **9**(4):e94476
28	GSE37180	Gene expression profiles of ovarian tumor biopsies from Phase I dasatinib trial	11	U133A2	Yes	Yes	No	No	No	NA	No	No	No	22837181	Secord AA, Teoh DK, Barry WT, Yu M et al. A phase I trial of dasatinib, an SRC-family kinase inhibitor, in combination with paclitaxel and carboplatin in patients with advanced or recurrent ovarian cancer. 2012 Clin Cancer Res **18**(19):5489-98.
29	GSE38734	Expression data from primary ovarian samples and matched abdominal deposits	4	Gene st1.0	No	No	No	No	No	NA	No	No	No	22896685	Cowin PA, George J, Fereday S, Loehrer E et al. LRP1B deletion in high-grade serous ovarian cancers is associated with acquired chemotherapy resistance to liposomal doxorubicin. 2012 Cancer Res **72**(16):4060-73.
30	GSE40595	A cancer associated fibroblasts (CAFs) specific gene signature in high grade serous ovarian cancer	32 + 6	U133P2	No	No	No	No	No	NA	No	No	Yes	23824740	Yeung TL, Leung CS, Wong KK, Samimi G et al. TGF-β modulates ovarian cancer invasion by upregulating CAF-derived versican in the tumor microenvironment. 2013 Cancer Res **73**(16):5016-28
31	GSE44104	COL11A1 promotes tumor progression and predicts poor clinical outcome in ovarian cancer.	60	U133P2	No	No	Yes	No	No	Serous/Endo/Clear/Muci	No	No	No	23934190	Wu YH, Chang TH, Huang YF, Huang HD et al. COL11A1 promotes tumor progression and predicts poor clinical outcome in ovarian cancer. 2014 Oncogene **33**(26):3432-40

32	GSE51373	Gene expression data from high grade serous ovarian cancer	28	U133P2	No	Yes	Yes	No	Yes	Serous	No	No	No		24237932	Koti M, Gooding RJ, Nuin P, Haslehurst A et al. Identification of the IGF1/PI3K/NF κB/ERK gene signalling networks associated with chemotherapy resistance and treatment response in high-grade serous epithelial ovarian cancer. 2013 BMC Cancer **13**:549
33	GSE55410	Gene expression analysis of samples from a phase II clinical trial of decitatbine in combination with carboplatin in patients with recurrent, platinum resistant epithelial ovarian cancer patients	13	Gene st1.0	No	No	No	No	Yes	Serous	No	No	No		20564122	Fang F, Balch C, Schilder J, Breen T, Zhang S, Shen C, Li L, Kulesavage C, Snyder AJ, Nephew KP, Matei DE. A phase 1 and pharmacodynamic study of decitabine in combination with carboplatin in patients with recurrent, platinum-resistant, epithelial ovarian cancer. 2010 Cancer **116**(17):4043-53
34	GSE32062	Immune-activation as a therapeutic direction for patients with high-risk ovarian cancer based on gene expression signature (1)	10	U133P2	Yes	Yes	No	No	No	Serous	No	No	No		22241791	Yoshihara K, Tsunoda T, Shigemizu D, Fujiwara H et al. High-risk ovarian cancer based on 126-gene expression signature is uniquely characterized by downregulation of antigen presentation pathway. 2012 Clin Cancer Res **18**(5):1374-85
35	GSE56443	Genomic analysis of low-grade serous ovarian carcinomas [EXP]	7	Gene st1.0	No	No	No	No	No	Low grade Serous	Yes	No	Yes		25316818	Emmanuel C, Chiew YE, George J, Etemadmoghadam D, Anglesio MS, Sharma R, Russell P, Kennedy C, Fereday S, Hung J, Galletta L, Hogg R, Wain GV, Brand A, Balleine R, MacConaill L, Palescandolo E, Hunter SM, Campbell I, Dobrovic A, Wong SQ, Do H, Clarke CL, Harnett PR, Bowtell DD, deFazio A; Australian Ovarian Cancer Study (AOCS). Genomic Classification of Serous Ovarian Cancer with Adjacent Borderline Differentiates RAS Pathway and TP53-Mutant Tumors and Identifies NRAS as an Oncogenic Driver. 2014 Clin Cancer Res. **20**(24):6618-30.
36	GSE62873	Late stage ovarian cancer gene expression profiles	63	Gene st1.0	No	No	Yes	Yes	No	Serous	No	No	No	---		---
37	GSE63885	Gene expression profiling in ovarian cancer	101	U133P2	Yes	Yes	Yes	No	No	Serous/Endo/Clear	No	Yes	No		24478986	Lisowska KM, Olbryt M, Dudaladava V, Pamuła-Piłat J et al. Gene expression analysis in ovarian cancer - faults and hints from DNA microarray study. 2014 Front Oncol **4**:6.
38	GSE30311/U133A	Kyoto Cohort (JPKO)	67	U133A	Yes	Yes	Yes	No	Yes	Serous/Endo/Clear/Muci	Yes	Yes	No		23078675	Huang RY, Chen GB, Matsumura N, Lai HC et al. Histotype-specific copy-number alterations in ovarian cancer. 2012 BMC Med Genomics **5**:47
39	GSE30311/GSE30274	Kyoto Cohort (JPKO)* Repeat	56	Gene st1.0	Yes	Yes	Yes	No	Yes	Serous/Endo/Clear/Muci	Yes	Yes	No		23078675	Huang RY, Chen GB, Matsumura N, Lai HC et al. Histotype-specific copy-number alterations in ovarian cancer. 2012 BMC Med Genomics **5**:47
40	GSE30311/GSE30284	TWTPO	42	Gene st1.0	No	Yes	No	No	No	Serous/Endo/Clear/Muci	No	No	No		23078675	Huang RY, Chen GB, Matsumura N, Lai HC et al. Histotype-specific copy-number alterations in ovarian cancer. 2012 BMC Med Genomics **5**:47
41	---	FRTLO	33	Gene st1.0	No	No	Yes	Yes	No	Serous	No	No	No		22687175	Malek JA, Martinez A, Mery E, Ferron G, Huang R, Raynaud C, Jouve E, Thiery JP, Querleu D, Rafii A. Gene expression analysis of matched ovarian primary tumors and peritoneal metastasis. 2012 J Transl Med. **10**:121.
42	---	OSLO	25	U133P2	Yes	Yes	Yes	Yes	No	Serous	No	Yes	No		23666744	Tan TZ, Miow QH, Huang RY, Wong MK, Ye J, Lau JA, Wu MC, Bin Abdul Hadi LH, Soong R, Choolani M, Davidson B, Nesland JM, Wang LZ, Matsumura N, Mandai M, Konishi I, Goh BC, Chang JT, Thiery JP, Mori S. Functional genomics identifies five distinct molecular subtypes with clinical relevance and pathways for growth control in epithelial ovarian cancer. 2013 EMBO Mol Med. **5**(7):983-98.
43	TCGA	The Cancer Genome Atlas	590	HT-U133A	Yes	Yes	Yes	No	Yes	Serous	No	Yes	No		21720365	Cancer Genome Atlas Research Network 2011 Nature **474** 60-15.
44	GSE9890	Expression profile of 5 ovarian tumour samples (two diff	5	U133P2	No	No	Yes	Yes	No	Serous	No	Yes	No		18698038	Tothill RW, Tinker AV, George J, Brown R et al. 2008 Clin Cancer Res**14** 5198-208.
45	GSE54807	RNA profiling from ovarian FFPE specimens	11	Gene st1.0	No	No	No	No	No	Serous/Clear	No	No	No	---		---
46	GSE69207	OvNUHsg	100	Gene st1.0	No	No	No	No	No	Serous/Clear/Endo	No	No	No	---		---
47	GSE52037	Transcriptional override: a regulatory network model of	10+10	U133P2	No	No	Yes	Yes	No	Serous	No	No	No		24666724	Hill CG, Matyunina LV, Walker D, Benigno BB et al. Transcriptional override: a regulatory network model of indirect responses to modulations in microRNA expression. 2014 BMC Syst Biol **8**:36
48	GSE23392	miRNAs in ovarian cancer: A systems approach	3+5	U133P2	No	No	Yes	No	Yes	Serous	No	Yes	Yes		21811625	Shahab SW, Matyunina LV, Mezencev R, Walker LD, Bowen NJ, et al. Evidence for the Complexity of MicroRNA-Mediated Regulation in Ovarian Cancer: A Systems Approach. 2011 PLoS ONE **6**(7): e22508.
49	GSE26193	Control of oxidative stress by miRNA and impact on ova	107	U133P2	Yes	Yes	Yes	Yes	No	Serous/Clear/Endo/Muci	No	Yes	No		22101765	Mateescu B, Batista L, Cardon M, Gruosso T et al. miR-141 and miR-200a act on ovarian tumorigenesis by controlling oxidative stress response. 2011 Nat Med **17**(12):1627-35

Abbreviation: Serous, high grade serous; clear, clear cell; Endo, Endometrioid; Muci, Mucinous; LMP, low malignant potential; NA, not applicable.
